# The Natural Product 6-Gingerol Inhibits Inflammation-Associated Osteoclast Differentiation via Reduction of Prostaglandin E_2_ Levels

**DOI:** 10.3390/ijms19072068

**Published:** 2018-07-16

**Authors:** Youn-Hwan Hwang, Taesoo Kim, Rajeong Kim, Hyunil Ha

**Affiliations:** Herbal Medicine Research Division, Korea Institute of Oriental Medicine, Daejeon 34054, Korea; hyhhwang@kiom.re.kr (Y.-H.H.); xotn91@kiom.re.kr (T.K.); younme1112@nate.com (R.K.)

**Keywords:** 6-gingerol, osteoclast, inflammation, interleukin-1, prostaglandin E_2_

## Abstract

The natural product 6-gingerol, a major bioactive component of the rhizome of ginger (*Zingiber officinale*), is known to have several beneficial effects on health, including anti-inflammatory activity. The present study aimed to investigate the effects of 6-gingerol on osteoclast differentiation associated with inflammation. 6-Gingerol inhibited osteoclast differentiation in co-cultures of osteoblasts and osteoclast precursor cells in response to the pro-inflammatory cytokine, interleukin (IL)-1. However, it did not affect osteoclast precursor differentiation into osteoclasts induced by the receptor activator of nuclear factor-κB ligand (RANKL), a key cytokine causing osteoclast differentiation. 6-Gingerol inhibited IL-1-induced RANKL expression in osteoblasts, and the addition of RANKL to the co-cultures overcame 6-gingerol-mediated inhibition of osteoclast differentiation. It also suppressed IL-1-induced prostaglandin E_2_ (PGE_2_) production in osteoblasts, and the addition of exogenous PGE_2_ reversed 6-gingerol-mediated inhibition of IL-induced RANKL expression in osteoblasts and osteoclast differentiation in the co-cultures. We found that 6-gingerol reduced PGE_2_ levels by suppressing enzymatic activities of cyclooxygenase and PGE synthase, which cooperatively catalyze the conversion of arachidonic acid to PGE_2_. Our findings demonstrate that 6-gingerol inhibits IL-1-induced osteoclast differentiation via suppression of RANKL expression in osteoblasts though reduction of PGE_2_ levels, suggesting its potential use in treating inflammatory bone destruction associated with excessive PGE_2_ production.

## 1. Introduction

Osteoclasts, the sole bone-resorbing cells, are multinucleated giant cells derived from hematopoietic monocyte–macrophage linage cells, and have a pivotal role in the pathogenesis of inflammatory bone disorders, such as rheumatoid arthritis, periodontal diseases, and periprosthetic osteolysis [1,2]. Under inflammatory conditions, excessive expression and production of osteoclastogenic mediators, including receptor activator of NF-κB ligand (RANKL) and macrophage colony-stimulating factor (M-CSF) from osteoblasts or stromal cells and inflammatory mediators from innate and adaptive immune systems, positively enhance osteoclast precursor formation, osteoclast differentiation, and bone resorption [3]. In particular, RANKL acts as an essential modulator of osteoclast differentiation and activation by directly binding to its receptor, RANK, expressed on osteoclast precursors and mature osteoclasts [4]. Thus, down-regulation of RANKL availability via control of RANKL expression and that of its decoy receptor, osteoprotegerin (OPG), has been shown to reduce osteoclast differentiation and bone loss in inflammatory conditions [5,6,7,8].

In addition to conventional drugs and therapies, several studies have investigated phytomedicines and nutraceuticals as treatments for overcoming excessive bone loss and abolishing undesirable and adverse effects of conventional drugs [9,10]. In east Asian, Ayurvedic, Arabic, and African traditional medicines, the rhizome of *Zingiber officinale* Roscoe (ginger) has been frequently used as a natural anti-inflammatory agent and pain-reliever in musculoskeletal diseases, such as arthritis, rheumatism, and muscular aches [11,12]. In recent human and experimental animal studies, the rhizome of *Z. officinale* has been demonstrated to be effective against inflammatory bone disorders, such as rheumatoid arthritis and osteoarthritis [13,14,15]. The rhizome of *Z. officinale* has a variety of pharmacological effects corresponding to several bioactive compounds, including the gingerols. In particular, 6-gingerol (C_17_H_26_O_4_; molecular weight, 294.39), a primary bioactive phenylpropanoid isolated from *Z. officinale*, has been shown to have anti-inflammatory, anti-oxidant, anti-tumoral, anti-diabetic, and anti-obesity activities in animal models [16]. 6-Gingerol-enriched products have shown improvement in joint inflammation in an experimental arthritis model due to their anti-inflammatory property [17]. Abusarah et al. [18] have demonstrated that 6-gingerol has a therapeutic effect in osteoarthritis via protection against oxidative stress and down-regulation of pro-inflammatory mediators in vitro and in vivo. It has also been shown to stimulate osteoblast differentiation and alleviate tumor necrosis factor (TNF)-α-induced suppression of osteoblast differentiation in osteoblast-like cells, suggesting its potential benefit in bone formation [19]. However, little is known about the effects of 6-gingerol on osteoclast differentiation under inflammatory conditions. In the present study, we aimed to investigate the effects of 6-gingerol on osteoclast differentiation; the cellular and molecular mechanisms underlying the effects of 6-gingerol on inflammation-associated osteoclast differentiation were also characterized.

## 2. Results and Discussion

### 2.1. Inhibitory Effects of 6-Gingerol on Osteoclast Formation via Down-Regulation of RANKL Expression

A variety of osteoclast differentiation assays have been developed using isolated primary cells and stable cell lines with various osteotropic factors, such as IL-1, lipopolysaccharides, parathyroid hormone, prostaglandin E_2_ (PGE_2_), M-CSF, and RANKL [20]. Among the assays, we selected two systems, the co-culture of primary mouse calvarial osteoblasts (POBs) and bone marrow cells (BMCs) with IL-1, and bone marrow-derived macrophage (BMM) cultures with M-CSF and RANKL, to discriminate between the indirect and direct effects of 6-gingerol on osteoclast differentiation. IL-1 is involved in osteoclast-mediated bone destruction under inflammatory conditions, including rheumatoid arthritis [3,21]. In this context, we used IL-1 to induce osteoclast differentiation in the co-culture system, in which IL-1 indirectly induces osteoclast differentiation of BMCs containing osteoclast precursors via its effects on POBs. In the BMM culture system, RANKL induces osteoclast differentiation via direct action on osteoclast precursors.

**Figure 1:**
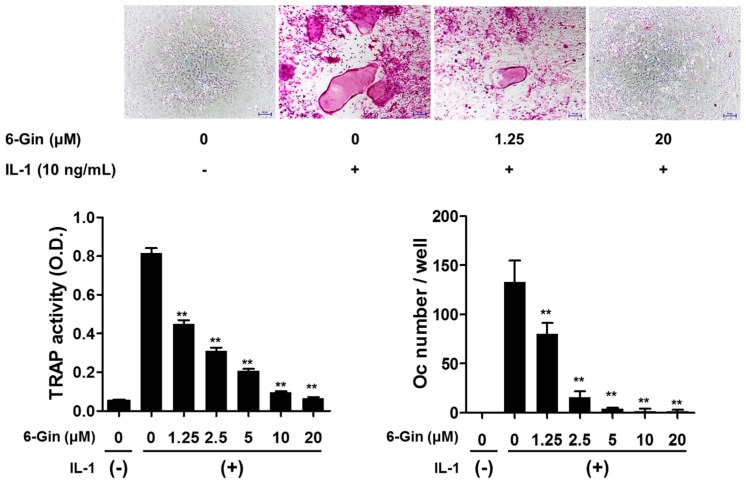
6-Gingerol inhibits IL-1-induced osteoclast formation in co-cultures of primary mouse calvarial osteoblasts (POBs) and bone marrow cells (BMCs). The co-cultures were incubated with IL-1 (10 ng/mL) for seven days with 6-gingerol (6-Gin, 1.25–20 μM) pretreatment 1 h prior to IL-1 treatment. After fixation, total cellular tartrate-resistant acid phosphatase (TRAP) activity was measured, and TRAP-positive multinucleated giant cells (≥three nuclei, ≥50 μm in diameter) were counted as osteoclasts. ** *p* < 0.01 vs. treatment with IL-1 alone. Scale bar = 100 μm.

**Figure 2:**
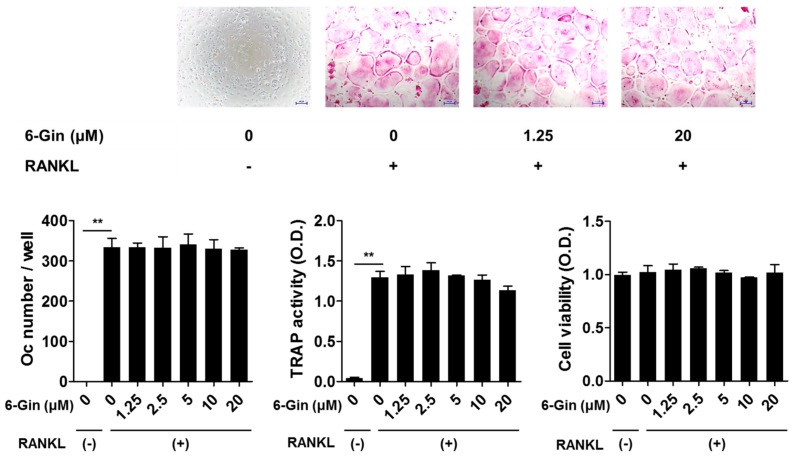
6-Gingerol does not affect receptor activator of NF-κB ligand (RANKL)-induced osteoclast formation in bone marrow-derived macrophages (BMMs). BMMs were incubated with or without macrophage colony-stimulating factor (M-CSF) (30 ng/mL), RANKL (100 ng/mL), and 6-gingerol (6-Gin, 1.25–20 μM) for four days. Total cellular TRAP activity and the number of osteoclasts were measured. Cell viability was determined using Cell Counting Kit-8 assay. ** *p* < 0.01 vs. control with no RANKL treatment. Scale bar = 100 μm.

Treatment with IL-1 for seven days increased tartrate-resistant acid phosphatase (TRAP) activity, a maker of osteoclast differentiation, and successfully induced osteoclast formation in co-cultures of POBs and BMCs, which was inhibited by 6-gingerol in a dose-dependent fashion (Figure 1). 6-Gingerol at concentrations higher than 10 μM caused almost complete inhibition of osteoclast formation. We next investigated whether 6-gingerol directly affected the differentiation of osteoclast precursors into osteoclasts. 6-Gingerol at concentrations up to 20 μM affected neither cell viability nor RANKL-induced osteoclast differentiation in BMM cultures (Figure 2). Based on the different effects of 6-gingerol in the two culture systems, we hypothesized that 6-gingerol affects the ability of POBs to support osteoclast differentiation.

**Figure 3:**
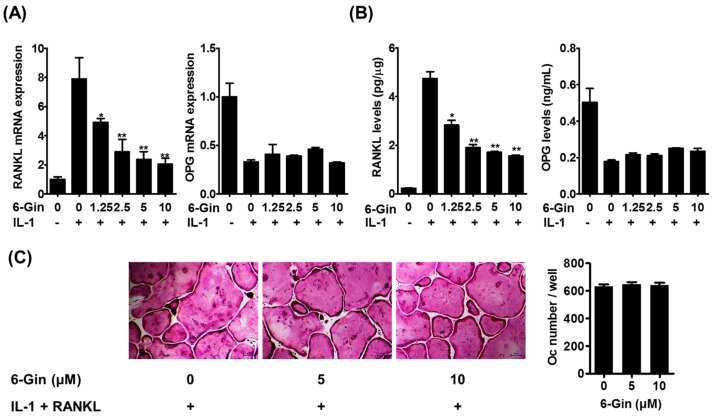
6-Gingerol inhibits IL-1-induced RANKL expression in POBs. (**A**) POBs were incubated with or without IL-1 (10 ng/mL) and 6-gingerol (6-Gin, 1.25–20 μM) for 24 h. Gene expression levels of RANKL and osteoprotegerin (OPG) were analyzed using quantitative real-time PCR; (**B**) RANKL levels in POB lysates and OPG levels in the culture medium were determined using corresponding enzyme-linked immunosorbent assay (ELISA) kits; (**C**) POBs and BMCs were co-cultured with or without 6-gingerol (5 or 10 μM), and IL-1 (10 ng/mL), and RANKL (50 ng/mL) for five days. The number of osteoclasts was counted. * *p* < 0.05 and ** *p* < 0.01 vs. treatment with IL-1 alone. Scale bar = 100 μm.

IL-1 is known to enhance the osteoclast-supporting activity of POBs by up-regulating RANKL expression and down-regulating expression of its decoy receptor, OPG [5,22]. Therefore, we examined whether 6-gingerol affects IL-1-induced RANKL and OPG expression in POBs. Real-time PCR analysis and enzyme-linked immunosorbent assay (ELISA) showed that IL-1 increased RANKL mRNA and protein expression in POBs; this was inhibited by 6-gingerol in a dose-dependent manner (Figure 3A,B). In contrast, 6-gingerol did not affect IL-1-induced reduction of OPG mRNA expression and protein secretion. The addition of exogenous RANKL to IL-1-stimulated co-cultures of POBs and BMCs reversed the inhibitory effects of 6-gingerol on osteoclast formation (Figure 3C). These results suggested that the anti-osteoclastogenic effects of 6-ginerol did not result from cytotoxicity in the co-cultures and that 6-gingerol-mediated suppression of RANKL expression in POBs contributed to its anti-osteoclastogenic effects in the co-cultures.

### 2.2. Inhibitory Effects of 6-Gingerol on IL-1-Induced PGE_2_ Production

**Figure 4:**
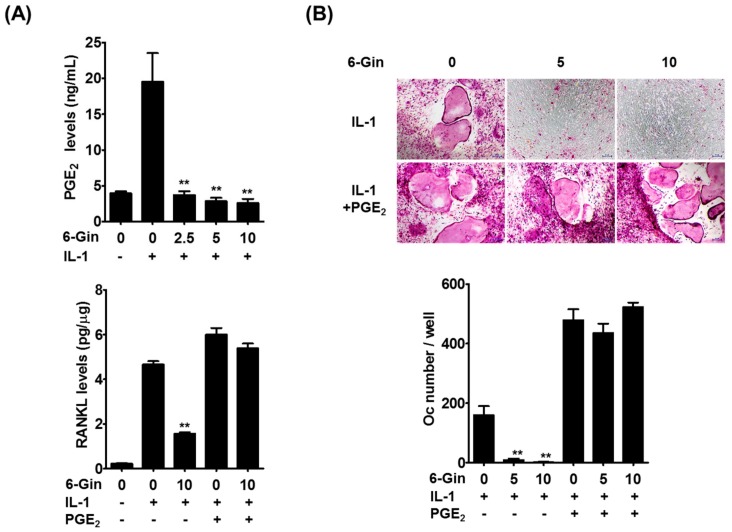
Prostaglandin E_2_ (PGE_2_) reverses the inhibitory effects of 6-gingerol on IL-1-induced osteoclast differentiation. (**A**) POBs were treated with or without IL-1 (10 ng/mL) and 6-gingerol (6-Gin, 2.5–10 μM) for 24 h. PGE_2_ levels in culture media and RANKL levels in cell lysates were determined; (**B**) the co-cultures of POBs and BMCs were incubated with or without 6-gingerol (5 or 10 μM), IL-1 (10 ng/mL), and PGE_2_ (100 nM) for seven days. The number of osteoclasts was counted. ** *p* < 0.01 vs. treatment with IL-1 alone. Scale bar = 100 μm.

We next sought to understand how 6-gingerol inhibits IL-1-induced RANKL expression in POBs. PGE_2_ is known to elevate RANKL expression in several cell types, including osteoblasts, and to induce osteoclast differentiation and osteoclast-mediated bone loss [7,23,24,25]. In addition, we previously found that PGE_2_ mediates IL-1-induced RANKL expression in POBs and osteoclast formation in co-cultures [5]. Therefore, we investigated whether PGE_2_ production was responsible for the inhibitory effects of 6-gingerol on IL-1-induced RANKL expression in POBs. IL-1 stimulated PGE_2_ production in POBs, which was inhibited by 6-gingerol. Inhibition of IL-1-induced RANKL protein expression by 6-gingerol was overcome by the addition of exogenous PGE_2_ (Figure 4A). In addition, exogenous PGE_2_ reversed the inhibitory effects of 6-gingerol on IL-1-induced osteoclast formation in the co-cultures (Figure 4B). These results strongly suggested that 6-gingerol inhibited IL-1-induced RANKL expression in POBs via suppression of PGE_2_ production, leading to inhibition of IL-1-induced osteoclast formation in the co-cultures.

**Figure 5:**
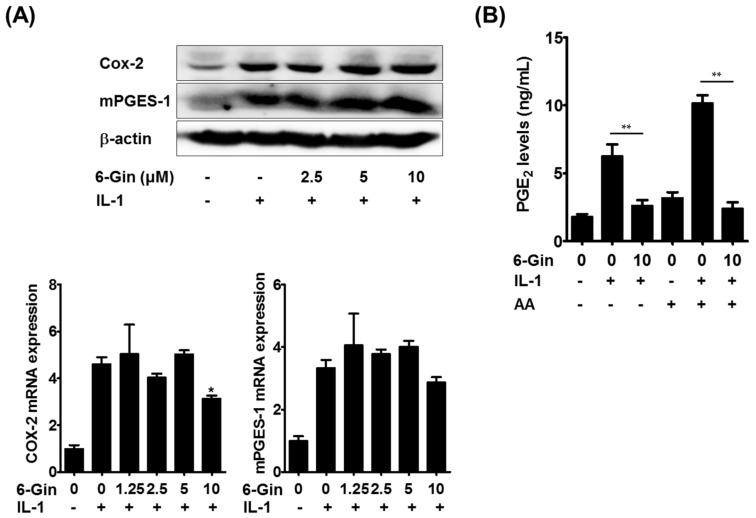
6-Gingerol inhibits IL-1-induced PGE_2_ synthesis without affecting the expression of COX (cyclooxygenase)-2 and microsomal PGES-1 (mPGES-1). (**A**) POBs were treated with or without 6-gingerol (6-Gin, 1.25–10 μM) and IL-1 (10 ng/mL) for 24 h. The protein and mRNA levels of COX-2 and mPGES-1 were determined via Western blot (upper panel) and real-time PCR (lower panel); (**B**) POBs were treated with or without IL-1 for 24 h, and the culture medium was replaced with fresh medium. Cells were then treated with or without 6-gingerol (10 μM) for 30 min and further incubated with or without arachidonic acid (AA, 5 μM) for 15 min. PGE_2_ concentrations in culture medium were determined using enzyme immunoassay (EIA). * *p* < 0.05 vs. treatment with IL-1 alone. ** *p* < 0.01.

We then attempted to elucidate the molecular mechanism(s) underlying 6-gingerol-mediated suppression of PGE_2_ production. PGE_2_ biosynthesis is achieved by sequential steps catalyzed by three groups of enzymes. These include the release of arachidonic acid from plasma membrane phospholipids by phospholipase A2 (PLA2) enzymes, the conversion of free arachidonic acid to PGH_2_ by cyclooxygenase (COX) enzymes, which is the rate-limiting step in the production of PGE_2_, and the final isomerization of PGH_2_ to PGE_2_ by PGE synthase (PGES) enzymes [26]. Among the isoenzymes involved in each step, cytosolic PLA2α (cPLA2), COX-2, and microsomal PGES-1 (mPGES-1) have been shown to be mainly involved in PGE_2_ synthesis in osteoblasts and stromal cells in response to IL-1 and LPS [5,24,27]. It was shown that 6-gingerol does not affect cPLA2 activity even at 10 μM [28]. Therefore, we examined whether 6-gingerol altered IL-1-induced expression of COX-2 and mPGES-1, which are highly induced in osteoblasts due to inflammatory stimuli [5,27]. Treatment of POBs with IL-1 for 24 h markedly increased the mRNA and protein expression of COX-2 and mPGES-1, which was not affected by 6-gingerol at concentrations below 5 μM (Figure 5A). Although 6-gingerol, at a concentration of 10 μM, slightly suppressed IL-1-induced COX-2 mRNA expression, it did not affect IL-1-induced up-regulation of COX-2 protein levels. Therefore, we next investigated whether 6-gingerol affected COX/PGES activity. In POBs pretreated with IL-1 for 24 h to induce COX-2 and mPGES-1 expression, 6-gingerol treatment for 30 min inhibited IL-1-indeuced PGE_2_ production. Furthermore, the addition of exogenous arachidonic acid further increased IL-1-induced PGE_2_ production, which was also abrogated by 6-gingerol (Figure 5B). These results suggested that 6-gingerol inhibited IL-1-induced PGE_2_ production by suppressing the COX/PGES-mediated conversion of arachidonic acid to PGE_2_. Extracellular PGE_2_ levels can also be regulated by other components of the COX pathway. Extracellular PGE_2_ is imported by the prostaglandin transporter and metabolized into an inactive form by 15-prostaglandin dehydrogenase [29]. In the present study, the addition of exogenous PGE2 completely reversed the inhibitory effects of 6-gingerol on IL-1-induced RANKL expression and osteoclast formation (Figure 4A,B), suggesting that the clearance of extracellular PGE_2_ was not mainly involved in 6-gingerol-induced reduction of extracellular PGE_2_ levels. However, further investigations are required to elucidate the detailed molecular mechanisms underlying 6-gingerol-mediated reduction of PGE_2_ levels.

In summary, we showed that 6-gingerol inhibits IL-1-induced osteoclast differentiation via suppression of RANKL expression in osteoblasts through reduction of PGE_2_ levels. In addition to such inhibitory effects on osteoclast differentiation, it has also been shown that 6-gingerol stimulates osteoblast proliferation and differentiation, and further restores osteoblast differentiation inhibited by the pro-inflammatory cytokine, TNF-α [19]. Our findings suggest that 6-gingerol may have beneficial effects on bone metabolism in inflammatory conditions.

## 3. Materials and Methods

### 3.1. Reagents and Antibodies

6-Gingerol and recombinant IL-1α were obtained from Sigma-Aldrich (St. Louis, MO, USA) and PeproTech (Rocky Hill, NJ, USA), respectively. Recombinant soluble RANKL was prepared as reported previously [30]. M-CSF was kindly provided by Yongwon Choi (University of Pennsylvania School of Medicine). α-Modified minimal essential medium (MEM) and fetal bovine serum (FBS) were obtained from Thermo Fisher Scientific Inc. (Rockford, IL, USA). Antibodies for COX-2 and mPGES-1 were purchased from BD Biosciences (Heidelberg, Germany) and Cayman Chemicals (Ann Arbor, MI, USA), respectively. β-actin antibody, rabbit anti-mouse IgG-horseradish peroxidase (HRP), and goat anti-rabbit IgG-HRP were obtained from Santa Cruz Biotechnology (Santa Cruz, CA, USA).

### 3.2. Cell Preparation and Osteoclast Formation Assays

The animal study was approved by the Institutional Animal Care and Use Committee of the Korea Institute of Oriental Medicine (permission numbers: 15-057 and 15-058, Daejeon, Korea). POBs were prepared from calvariae of newborn Institute of Cancer Research (ICR) mice (Samtako, Osan, South Korea) as described previously [31]. BMCs were obtained from the femurs of male ICR mice (5–7 weeks old), and BMMs were prepared from BMCs using M-CSF as described previously [31]. To determine the effects of 6-gingerol on osteoclast differentiation, we employed two different culture systems as follows; (1) co-cultures of POBs and BMCs; and (2) BMM cultures treated with M-CSF and RANKL. First, POBs (2.5 × 10^4^ cells) and BMCs (3 × 10^5^ cells) were co-cultured with or without IL-1 (10 ng/mL), RANKL (50 ng/mL), and PGE_2_ (100 nM) in α-MEM medium containing 10% FBS in a 48-well tissue culture plate. 6-Gingerol pre-treatment was done 1 h prior to IL-1 exposure. For the other osteoclast formation assay, BMMs (1 × 10^4^ cells/well) were cultured for four days in the presence of M-CSF (30 ng/mL) and RANKL (100 ng/mL) with or without 6-gingerol in a 96-well plate. Total cellular TRAP activity was measured colorimetrically with *p*-nitrophenyl phosphate as a substrate. TRAP staining was carried out using naphthol AS-MX phosphate and fast red violet LB salt as described previously [32]. TRAP-positive multinucleated (≥three nuclei and ≥50 μm of diameter) cells were considered to be osteoclasts. Cytotoxicity was measured using a Cell Counting Kit-8 per the manufacturer’s instructions (Dojindo Molecular Technologies Inc., Rockville, MD, USA)

### 3.3. Measurement of RANKL, OPG, and PGE2 in POBs

POBs (3 × 10^4^ cells/well) were cultured with or without IL-1 (10 ng/mL) for 24 h in a 12-well culture plate. Before IL-1 treatment, 6-gingerol treatment at concentrations of 1.25–10 μM was done. Levels of RANKL in POB lysates and those of OPG in culture media were measured using corresponding ELISA kits (R&D systems, Minneapolis, MN, USA). PGE_2_ levels in culture supernatants were determined using an enzyme immunoassay (EIA) kit (Cayman Chemicals, Ann Arbor, MI, USA).

### 3.4. Measurement of COX/PGES Activity in POBs

POBs were pretreated with or without IL-1 (10 ng/mL) for 24 h to induce COX-2 and mPGES-1 expression, and the culture medium was replaced with fresh medium containing different concentrations of 6-gingerol. After 30 min of incubation, arachidonic acid (5 μM) was added for 15 min, and PGE_2_ levels in the culture medium were then measured as above.

### 3.5. Quantitative Real-Time PCR

Total RNA was extracted using the RNeasy kit (Qiagen, Hilden, Germany), and cDNA was synthesized from 1 μg of total RNA using the High-Capacity cDNA Reverse Transcription Kit (ABI, Waltham, MA, USA), per the manufacturer’s instructions. For real-time PCR analysis, an ABI 7500 Real-Time PCR System was used in combination with TaqMan probes (Thermo Scientific, Rockford, IL, USA) and the TaqMan Universal Master Mix. In POBs, mRNA levels of *RANKL* (Mm0041908_m1), *OPG* (Mm00435452_m1), *COX-2* (Mm00478374_m1), and *mPGES-1* (Mm00452105_m1) were estimated, and the experiments were repeated in triplicate. The 18S ribosomal gene (Hs99999901_s1) was used to normalize mRNA expression levels, and the relative expression was calculated using the ΔΔ*C*t method.

### 3.6. Western Blot Analysis

Protein contents in the cell lysates prepared using RIPA buffer were determined using a bicinchoninic acid assay kit (Thermo Scientific). Total protein (40 μg) was resolved using 12.5% SDS-PAGE gel electrophoresis and transferred to polyvinylidene fluoride membranes. The membranes were blocked with 5% bovine serum albumin and incubated overnight at 4 °C with primary antibodies against COX-2 (1:1000), mPGES-1 (1:500), and β-actin (1:1000). The blots were then incubated with HRP-conjugated secondary antibodies and visualized using a chemiluminescence reagent (Thermo Scientific).

### 3.7. Statistical Analysis

All data were represented as mean ± standard deviation. Experiments were repeated three times, and results from one representative experiment are shown. Data were subjected to a Student’s t-test or one-way analysis of variance followed by a Dunnett’s test using the software Prism (version 5.0) (San Diego, CA, USA), and a value of *p* < 0.05 was considered statistically significant.

## 4. Conclusions

In the present study, we investigated the effects of 6-gingerol on osteoclast differentiation and the underlying molecular mechanisms. We show that 6-gingerol inhibits IL-1-induced osteoclast differentiation through down-regulation of RANKL expression in osteoblasts by suppressing PGE_2_ synthesis. Given the important roles of PGE_2_ in inflammatory diseases, the inhibitory action of 6-gingerol on PGE_2_ synthesis may provide a molecular basis for its anti-inflammatory effects and its potential use in treating inflammatory bone loss.
